# Vertigo and Severe Balance Instability as Symptoms of Lyme Disease—Literature Review and Case Report

**DOI:** 10.3389/fneur.2019.01172

**Published:** 2019-11-12

**Authors:** Magdalena Jozefowicz-Korczynska, Ewa Zamyslowska-Szmytke, Anna Piekarska, Oskar Rosiak

**Affiliations:** ^1^Balance Disorders Unit, Department of Otolaryngology, Medical University of Lodz, The Norbert Barlicki Memorial Teaching Hospital, Lodz, Poland; ^2^Nofer Institute of Occupational Medicine, Balance Disorders Unit, Department of Audiology and Phoniatrics, Lodz, Poland; ^3^Department of Infectious Diseases and Hepatology, Medical University of Lodz, Lodz, Poland

**Keywords:** vertigo, Lyme disease, neuroborreliosis, vestibular rehabilitation, dizziness

## Abstract

Lyme disease is caused by a tick-borne bacterium *Borrelia* sp. This zoonotic infection is common in the Northern Hemisphere, e.g., Europe. Clinical presentation may involve multisystem symptoms and depends on the stage of the disease. The involvement of nervous system in Lyme disease is commonly referred to as neuroborreliosis. Neuroborreliosis may involve meningitis, mononeuritis multiplex, or cranial neuritis including the inflammation of vestibulocochlear nerve. In the late or chronic stage of Lyme disease, vestibular involvement may be the sole presentation, although such cases are rare. Our study was designed to present our own case and review the available literature reporting cases of neuroborreliosis with vertigo/dizziness and severe balance instability as a main disease symptom. The studies were obtained by searching the following databases: PubMed, Medline, and Embase. We included case reports of Lyme disease presenting with vertigo or gait disorders as the main symptom, written in the English language. Initially, 60 papers were identified. After analyzing the abstracts, seven manuscripts focusing on 13 clinical cases were included in this review. We conclude that the patients with neuroborreliosis sometimes present vertigo/dizziness, but rarely gait ataxia as a sole symptom. These complaints are usually accompanied by a hearing loss. Antibiotic treatment is usually effective. Balance instability in the patients with neuroborreliosis may persist but it responds well to vestibular rehabilitation.

## Introduction

Lyme disease, caused by *Borrelia* sp. spirochete, is a zoonotic infection that spreads through tick bites. In Europe, the natural carrier for *Borrelia burgdorferi*, which is the most common species responsible for Lyme borreliosis, is a tick *Ixodes ricinus*. Epidemiological data suggest that the number of cases in Europe has increased steadily over the past two decades, reaching a total of 360,000 reported cases (1). Clinical manifestations of Lyme disease depend on its stage: early and late. The central nervous system is involved in ~10–15% of Lyme disease patients and is referred to as neuroborreliosis. About 5% of infected individuals develop early onset neuroborreliosis that starts to be noticeable 4–6 weeks following tick bite, whereas over 95% of infected patients develop neurological symptoms as late as 6 months from the initial infection (2). The patients with neuroborreliosis usually develop meningitis, mononeural neuritis, and cranial nerve palsy. Typically, if cranial nerves are involved, it is the facial nerve that is most affected. However, there were isolated reports describing cases of vestibulocochlear nerve involvement with hearing loss or vertigo (3, 4). Sole involvement of the vestibular system might present as dizziness and balance instability, whereas involvement of the cochlear nerve may be manifested by tinnitus and hearing loss. Gait ataxia is a very rare presentation and is usually accompanied by other symptoms. Such clinical manifestation can mimic a cerebellopontine lesion, and initially, such patients are often referred to neurologists and otorhinolaryngologists.

The diagnosis of neuroborreliosis may be problematic because the patients often do not recall being bitten by a tick. Additionally, if the bite occurred in a concealed area, there is a chance that the typical skin lesion (erythema) might not have been noticed. The diagnostic methods can be split into direct and indirect ones. Direct identification of pathogen involves diagnostic PCR with the cerebrospinal fluid (CSF) and material obtained from the skin lesion. The second direct diagnostic method is the bacterial culture. Unfortunately, the Borrelia culture is lengthy (up to several weeks) and still not internationally standardized. Indirect diagnosis involves identification of patient's antibodies directed against *Borrelia* sp. Two-tiered serological methods are usually used, the first being enzyme-linked immunoassay and the second being confirmatory test with use of Western blotting (or immunoblotting) technique. Detection of serum antibodies against *Borrelia* is a highly sensitive method but still not free of producing false-positive results, for instance due to infection with other bacteria, such as *Treponema pallidum* or *Helicobacter pylori* (5).

Bacterial infections of the nervous system are highly responsive to antimicrobial therapy; however, in rare cases, some symptoms may persist. The existence of chronic borreliosis or post-treatment Lyme disease syndrome has been the subject of debate in recent years (6).

In the present study, we review the available literature and summarize the case reports of neuroborreliosis with accompanying vertigo and balance instability and present one case report from our own clinical practice.

## Case Description

A 46-year-old male farmer was initially admitted to the Department of Neurology in 2018 with a sudden onset of tinnitus and hearing loss in the left ear, dizziness, severe balance instability, and gait ataxia. Neurological examination revealed no changes. Initially, a vestibular schwannoma was suspected, but the diagnostic imaging (CT, MRI, and angio-CT) revealed no pathologies. In addition, Doppler ultrasonography revealed no disturbances in a blood flow in vertebral or carotid arteries. However, lumbar puncture revealed an increased protein concentration (47 mg/dl) and cytosis (6.0/μl) in the CSF. Patient serum and CSF were tested for IgG and IgM *Borrelia*-specific antibodies using ELISA (LIAISON Borrelia, Manufacturer: DiaSorin). Positive results in ELISA were then confirmed with Western blot test (EUROLINE-RN-AT-adv for IgM and EUROLINE-RN-AT for IgG, Manufacturer: EUROIMMUN). Analysis by Western blot was positive for IgG antibodies in the CSF and serum. The antibody index (AI = QBb/QIg) was calculated from the ratio between CSF/serum for specific antibodies (QBb) and total immunoglobulins (QIg) and was negative for intrathecal antibody synthesis.

**Figure 1:**
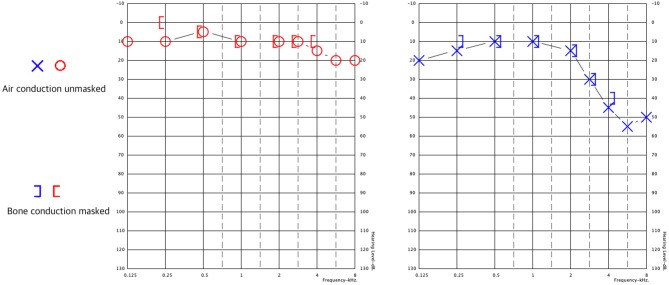
Pure tone audiometry results. Right ear in red, left ear in blue. Both ears exhibit sensorineural hearing loss at 3–8 kHz, which is greater in the left ear.

Following diagnosis, a 3-week course of doxycycline was initiated. Despite therapy, dizziness and gait impairment persisted. The patient was referred to the Balance Disorders Unit for vestibular evaluation. Audiometry revealed mild SNHL (sensory-neural hearing loss) in the right ear and mild to moderate SNHL for higher-frequency sounds, which was greater in the left ear. Speech discrimination was worse for the left ear (Figure 1) than the right ear (100% at 60 dB vs. 100% at 30 dB).

Upon examination, the patient presented with severe gait disturbance and imbalance. Romberg's test was positive. Unterberger sign was positive to the left side. Static and dynamic post-urography results were abnormal and the caloric test performed with videonystagmography (Ulmer SYNAPSIS 2008) determined canal paresis (87% left) with directional preponderance (7% right), and in addition, the saccadic pursuit eye movements and optokinetic tests were abnormal, confirming unilateral weakness on the left side and central vestibular system impairment. Clinical tests including Dynamic Gait Index (DGI), Berg Balance Scale (BBS), and Tinnetti test were conducted before and after rehabilitation (Table 1).

**Table 1:** Results of clinical tests and post-urography before and after vestibular rehabilitation therapy.

**Test**	**Before rehabilitation**	**After rehabilitation**
DGI (total score in pts.)	13	19
BBS (total score in pts.)	40 (medium risk of fall)	50 (low risk of fall)
Tinnetti (total score in pts.)	19 (moderate risk of fall)	24 (low risk of fall)
Static post-urography (total COM path length in mm) quiet stance, eyes open	183	92
Static post-urography (total COM mean velocity in mm/s) quiet stance, eyes open	0.73	0.55
Static post-urography (total COM path length in mm) quiet stance, eyes closed	314	201
Static post-urography (total COM mean velocity in mm/s) quiet stance, eyes closed	1.57	0.9
Dynamic post-urography SOT score	30	52

*COM, center of mass; SOT, Sensory Organization test; DGI, Dynamic Gait Index; BBS, Berg Balance Scale*.

The patient underwent 10 consecutive, daily, 30-min sessions of vestibular rehabilitation therapy {individual rehabilitation program consisting of Cawthorne–Cooksey exercises and virtual reality rehabilitation [as per protocol described in our previous study (7)]} with significant balance improvement in eyes-open conditions. Improvement in DGI, BBS, and Tinnetti score was noted, shifting the patient from moderate to low risk of fall.

In control post-urography, a decrease in total length and mean velocity of COM was visible as well as an improvement in SOT score (30 vs. 52). The improvement in static post-urography was greater for eyes-closed conditions. The patient remains under close observation; tinnitus and hearing loss remained. The patient was not fitted with a hearing aid to the left ear because of satisfactory speech discrimination threshold.

## Literature Review

### Materials and Methods

The literature was collected during a comprehensive search of using the online databases PubMed, Medline on Ovid, and Embase on Ovid up until September 2019. The following search string was used: (Lyme Neuroborreliosis[Mesh] OR Neuroborreliosis[tw] OR “Nervous System Lyme Borreliosis”[tw] OR “Peripheral Nervous System Lyme Disease”[tw] OR “Lyme Meningoradiculitis”[tw] OR “Lyme Polyradiculitis”[tw] OR “Lyme Polyradiculopathy”[tw] OR “Central Nervous System Lyme Disease”[tw] OR “Lyme Meningoencephalitis”[tw] OR Lyme disease[mh] OR “Lyme disease”[tw] OR B. burgdorferi Infection[tw] OR “Lyme Borreliosis”[tw] OR “Borrelia burgdorferi Infection”[tw] OR “Lyme Arthritis”[tw]) AND (Vertigo[Mesh] OR Vertigo[tw] OR Dizziness[Mesh] OR Dizziness[tw] OR Orthostasis[tw] OR Lightheadedness[tw] OR vestibular[tw] OR Light-Headedness[tw] OR “Light Headedness”[tw] OR “unsteady gait”[tw] OR Gait Ataxia[Mesh]).

In our review, the following inclusion criteria were established: (1) subject had to be human; (2) case report should provide a thorough patient history and laboratory findings including serum and CSF *Borrelia* antibody tests; (3) main complaint was vertigo or gait disturbance/ataxia; and (4) only full text studies written in English that were published in peer-reviewed journals were included in further analysis.

Two independent reviewers analyzed the abstracts and identified the papers meeting the inclusion and exclusion criteria. The cases were extracted from full-text manuscripts and summarized according to their clinical and laboratory findings. Furthermore, we include one case of neuroborreliosis with vertigo and gait ataxia diagnosed in our unit.

### Results

Initial search result returned 60 non-duplicated results. Forty-three full-text articles were available in English. Six manuscripts were excluded because final diagnosis was different from Lyme disease, 8 studies reported other symptoms outside the scope of this analysis, 12 manuscripts presented pooled data, 2 were experimental studies, 2 were reviews, and 4 manuscripts not reporting CSF testing were excluded from further analysis. Review of full-text articles identified seven manuscripts describing case reports that met the inclusion criteria and one retrospective study reporting detailed results of eight individual cases of Lyme disease with vertigo, two of which were excluded due to lack of CSF testing. Clinical data were extracted by reviewers from the full-text manuscripts and are summarized in Table 2 together with data from the case report described above.

**Table 2:** Summary of clinical and laboratory results in patients with neuroborreliosis presenting vertigo as the main symptom.

**Patient age/sex**	**Study**	**Symptoms**	**Days to diagnosis**	**Borrelia antibody testing**		**Otological diagnostics**	**Outcome**
				**Serum**	**CSF**		
46/M	Own case report	V, HL, T, I	10	IgM (–) IgG (+)	IgG (+)	SNHL VNG ab. POST ab.	V, I Imp. T, HL Per.
58/F	Huda et al. (8)	V, HL, I,OS	450	(–)	IgG (+) IgM (+)	SNHL	All.
62/M	Peltomaa et al. (9)	V, HL	90	IgM IgG (+)	(–)	SNHL	Sub.
50/F	Peltomaa et al. (9)	V, OS	284	IgG (+)	(–)	Audiometry no. POST no. ENG no.	H, A sub. V Imp.
52/F	Peltomaa et al. (9)	V, T	192	IgG (+)	(–)	SNHL POST ab. Caloric test ab.	Sub.
8/F	Peltomaa et al. (9)	V, T	150	IgM (+) IgG (+)	(–)	Audiometry no. POST ab.	Sub.
57/F	Peltomaa et al. (9)	V, T, HL	60	IgG (+)	(–)	ENG no. SNHL	H, V Imp. T, I Per.
38/F	Peltomaa et al. (9)	V,T,HL,OS	90	IgG (+)	(–)	SNHL ENG no.	Sub.
15/M	Curless et al. (10)	V, HL	30	IgG (–) IgM (–)	IgM (+)	N/A	Sub.
49/M	Ishizaki et al. (11)	V, T	30	IgG (+)	(–)	ENG ab. Caloric test no.	Sub.
12/M	Heininger et al. (12)	V, OS	7	IgM (+)	IgM (+)	ENG ab.	Sub.
69/M	Leeuwen et al. (13)	V, I	90	IgM (+) IgG (+)	(–)	VNG ab.	Sub.
28/F	Farshad et al. (14)	V, I	42	IgG (+) IgM (+)	IgG (+) IgM (+)	N/A	Sub.
80/F	Aboul-Enain et al. (15)	I, OS	N/A	IgG (–) IgM (–)	IgG (+) IgM (+)	N/A	Sub.

*V, vertigo; T, tinnitus; HL, hearing loss; I, instability; OS, other symptoms; N/A, no data; SNHL, sensorineural hearing loss; no, normal; ab, abnormal; (+), positive; (–), negative; POST, posturography; Sub., Subsided; Imp., improved; Per., persistent*.

## Discussion

We describe here a case that presented with neurological symptoms such as vertigo, gait ataxia, hearing impairment, and tinnitus. In addition, the patient had pathological parameters in the CSF and positive tests for *Borrelia*-specific IgG antibodies in the CSF and serum. According to the European Federation of Neurologic Societies (EFNS), at least two of the following diagnostic criteria should be met to consider possible neuroboreliosis and three for a definite neuroboreliosis: (i) neurological symptoms, (ii) CSF pleocytosis, and (iii) intrathecal *Borrelia*-specific antibodies (16). Thus, our patient fulfills the criteria for possible neuroboreliosis. Unfortunately, the antibiotic therapy was not successful in decreasing hearing loss or tinnitus, suggesting permanent damage to the hearing nerve and cochlea.

All cases extracted from the literature had vertigo or dizziness, which may be considered a neurological manifestation of neuroborreliosis and therefore fulfilling the first diagnostic criterion. The presence of anti-*Borrelia*-specific antibodies in the CSF was reported only in six cases, whereas in eight cases, no Bb antibodies in the CSF were detected. It should be mentioned that only three case reports (8, 13, 14) were published after the EFNS guidelines were introduced in 2010. Gait ataxia manifesting as complete inability to walk is very rare, and has only been reported in our case and one other case in literature, which did not report CSF antibody testing (17).

Despite not reaching a definite diagnosis of neuroborreliosis, in most cases, symptoms subsided after a course of antibiotic treatment. A course of oral or intravenous antibiotic as per EFNS guidelines should be considered in cases suspected of Lyme disease with solely vestibular manifestations, which fulfill at least the criteria for possible neuroborreliosis. Diagnostic imaging by MRI is highly recommended to exclude possible vestibular schwannoma or internal auditory canal tumors, which might account for similar symptoms (9).

## Conclusions

Our review demonstrates that vertigo and gait ataxia may be a symptom of Lyme disease in some cases. Furthermore, antibiotic treatment of borreliosis is usually effective in reducing the otoneurological symptoms. Some balance instability may persist but responds well to the subsequent vestibular rehabilitation.

## Ethics Statement

Ethical review and approval was not required for the study on human participants in accordance with the local legislation and institutional requirements. The patients/participants provided their written informed consent to participate in this study. Written informed consent was obtained from the individual(s) for the publication of any potentially identifiable images or data included in this article.

## Author Contributions

MJ-K: conceived and designed the analysis, wrote the paper. EZ-S: contributed data, performed post-urographic assessments. AP: contributed data, analyzed laboratory results, participated in treatment. OR: collected the data, performed the analysis.

### Conflict of Interest

The authors declare that the research was conducted in the absence of any commercial or financial relationships that could be construed as a potential conflict of interest.
